# BMP6 knockdown enhances cardiac fibrosis in a mouse myocardial infarction model by upregulating AP‐1/CEMIP expression

**DOI:** 10.1002/ctm2.1296

**Published:** 2023-06-14

**Authors:** Guiping Lu, Zhuowang Ge, Xinyuan Chen, Yue Ma, Ancai Yuan, Yuquan Xie, Jun Pu

**Affiliations:** ^1^ Department of Cardiology Renji Hospital Affiliated to Shanghai Jiao Tong University School of Medicine Shanghai China; ^2^ Department of Cardiology Xinhua Hospital Affiliated to Shanghai Jiao Tong University School of Medicine Shanghai China

**Keywords:** bone morphogenetic protein 6, cardiac fibrosis, myocardial infarction

## Abstract

**Background:**

The cardiac repair process following a myocardial infarction is a key factor in patient prognosis. In this repair process, cardiac fibrosis takes a critically important role. Among those featured genes for fibrosis, transforming growth factor beta (TGF‐β) is known to be involved in the fibrosis in various organs. And bone morphogenetic protein (BMP)6 belongs to the TGF‐β superfamily. Although BMPs are known to play exclusive roles in cardiac repair processes, the character of BMP6 in cardiac remodelling remains unclear.

**Purpose:**

This study aimed to investigate how BMP6 functioned in cardiac fibrosis following myocardial infarction (MI).

**Results:**

In this paper, we demonstrated that BMP6 expression was upregulated after myocardial infarction in wild‐type (WT) mice. Furthermore, BMP6^−/−^ mice showed a more significant decline in cardiac function and lower survival curves after MI. An enlarged infarct area, increased fibrosis and more pronounced inflammatory infiltration were observed in BMP6^−/−^ mice compared to WT mice. The expression of collagen I, collagen III and α‐SMA was increased in BMP6^−/−^ mice. In vitro, through gain‐of‐function and loss‐of‐function experiments, it was demonstrated that BMP6 decreases collagen secretion in fibroblasts. Mechanistically, knocking down BMP6 promoted AP‐1 phosphorylation, which in turn promotes CEMIP expression, led to an acceleration in the progression of cardiac fibrosis. Finally, it was found that rhBMP6 would alleviate ventricular remodelling abnormalities after myocardial infarction.

**Conclusion:**

Therefore, BMP6 may be a novel molecular target for improving myocardial fibrosis and cardiac function after myocardial infarction.

## Graphical abstract

- Knocking out BMP6 promotes AP‐1 phosphorylation in hypoxia‐induced cardiac fibroblasts.
- The expression of CEMIP is upregulated post‐MI, which is affected by AP‐1/BMP6.
- rhBMP6 affects the process of cardiac remodeling, which may be a potential therapeutic target in the cardiac remodeling post‐MI.

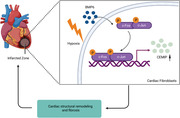

## 1 INTRODUCTION

The mortality of myocardial infarction (MI) due to coronary artery disease has declined globally with scientific advance and lifestyle change,^1^ primarily as a result of a decreased incidence of MI.^2^ However, when MI occurs, there are a number of potential outcomes, including early inflammation in acute MI, aseptic inflammation due to myocyte death, poor ventricular remodelling after myocardial infarction, limited cardiac systolic–diastolic function after myocardial fibrosis, scar formation and even cardiac rupture.^3^ Among these outcomes following myocardial infarction, poor ventricular remodelling played a key role, of which the primary factor influencing this process is fibroblast activation. Increased myocardial fibroblasts secrete excessive collagen and excessive deposition of extracellular matrix, which leads to myocardial fibrosis.^4^

BMPs are members of the transforming growth factor beta (TGF‐β) superfamily originally identified as osteo‐inducible proteins,^5^ which also function as cellular secretory factors. And there have been over 20 types of BMPs already identified to date. These known BMPs have been further subclassified based on protein function and nucleotide similarity (i.e., BMP2/4, BMP5/6/7/8, BMP9/10 and BMP12/13/14^6^). BMPs have been shown to play a significant role in embryonic heart development. For example, a knockdown of BMP2 resulted in abnormal heart development in mice.^7^ In addition, recent studies support the protective role of BMP signalling during post‐infarction remodelling, including the mutual antagonism between BMP2 and BMP4 in post‐infarction ventricular remodelling.^8^ Moreover, BMP7 has been shown to inhibit TGF‐β‐mediated fibrosis gene expression by activating SMAD‐1/5/8.^9^ The levels of BMP6 protein expression were elevated in patients with advanced heart failure, indicating that BMP6 is a promising cardiac marker to predict heart failure and a possible protective factor to the various subsequent cardiac pathophysiological changes following heart failure.^10^ Nevertheless, the role of BMP6 in coronary heart diseases, especially cardiac remodelling after MI, still remains largely unknown.

Among other diseases, the study by Lian et al. reported that BMP6 downregulation enhanced breast cancer cell proliferation.^11^ BMP6 and BMP7 inhibited estrogen‐induced breast cancer cell proliferation, which could be accomplished by inhibiting p38 mitogen‐activated protein kinase activation.^12^ Additionally, Arndt et al. demonstrated that BMP6 played a protective role in patients with NAFLD by inhibiting liver fibrosis.^13^ Moreover, it was reported that recombinant BMP6 could reverse the pro‐fibrotic effect of TGF‐β on HK‐2 cells.^14^ Present study also showed that a knockdown of BMP6 induced increased activating protein‐1 (AP‐1) activity, which critically exacerbated skin fibrosis.^15^ Transcriptional protein AP‐1 is a homodimer or heterodimer synthesized from various subunits, including Fos, Jun and ATF.^16^ In addition, the stimulation of AP‐1 transcriptional activity induces cellular proliferation, differentiation and apoptosis. By contrast, the inhibition of AP‐1 signalling eliminates fibroblast activation, thereby preventing experimental fibrosis.^17^ However, the function and specific mechanisms of AP‐1 as well as BMP6 in cardiac fibrosis have not been fully elaborated yet.

Therefore, the purpose of this study was to clarify the role of BMP6 in cardiac remodelling and to further elucidate the mechanisms involved in its influence on ventricular remodelling following MI.

## 2 MATERIALS AND METHODS

### 2.1 Animals

Six‐ to eight‐week‐old male C57BL/6J mice were purchased from Spelford (Beijing, China). BMP6 knockout male mice (Strain No. T010564), 6−8 weeks old, were obtained by CRISPR–Cas9 system on the C57BL/6JGpt genetic background, which were purchased from GemPharmatech (Nanjing, China). All mice were fed with normal chow and water. Mouse genotypes were determined by one‐step mouse genotyping kit (Vazyme, PD101‐01). All animal experiments were approved by the Animal Ethics Committee of Renji Hospital, School of Medicine, Shanghai Jiao Tong University.

### 2.2 Western blot

RIPA (strong) protein lysate was added to the cells or heart tissue. Then the samples were placed on ice for 10 min. After 10 min, the samples were centrifuged at 16 000 × *g* for 30 min. Then, the supernatant was collected as it contained the protein.^18^ The BCA method was used to detect protein concentration, which was calculated depending on the final protein standard curve. Generally, myocardial tissue protein concentration is quantified to 3 mg/mL. Five times the loading buffer was added to the samples, which was boiled at 100°C for 10 min. Proteins of different molecular values were separated through sodium dodecyl sulfate‐polyacrylamide gel electrophoresis (SDS‐PAGE). After transferring protein to PVDF membranes and being blocked in 5% skimmed milk powder or bovine serum albumin (BSA), different PVDF membranes were incubated with corresponding primary antibody at 4°C overnight. An antibody dilution was used to dilute the primary antibodies. BMP6 (abclonal, A4538), collagen I (servicebio, GB113041) and collagen III (servicebio, GB11023) antibodies were diluted at a 1:1000 ratio, and α‐SMA (servicebio, GB111364) was diluted at 1:10 000. The dilution of CEMIP antibody (proteintech, Cat No. 21129‐1‐AP) was 1:800. The secondary antibody was added to incubate PVDF membranes at room temperature for 1 h on alternate days. The chemiluminescent solution was used to visualize the protein expression in accordance with the manufacturer's instructions.

### 2.3 Myocardial infarction model

First, mice were anaesthetized with isoflurane before fixing the limbs in the supine position. Then, a vertical incision was made in the neck to expose the trachea for tracheal intubation. Isoflurane inhalation was maintained to keep the mice under anaesthetic state. After cutting the skin in the left anterior chest, the pectoralis major muscle was separated to expose the third intercostal space. The heart could be observed by breaking the intercostal space. Finally, coronary artery ligation was achieved using a suture with thread.^19^ Successful ligation is observed when the myocardial tissue beneath the ligation site is whitened. Finally, the skin was sutured.

### 2.4 RNA isolation and RT‐qPCR

As mentioned previously,^20^ the total cellular or cardiac tissue RNA was extracted using Trizol reagent (Vazyme, R401‐01). Then, the RNA was reverse‐transcribed to cDNA using a reverse transcription kit (YEASEN, 11141ES60). PCR amplification was performed after the reaction system was configured using a quantitative real‐time PCR kit (YEASEN, 11202ES08) in accordance with the manufacturer's instructions.

### 2.5 Cardiac fibroblast isolation and culture

After disinfecting the skin of the C57BL/6J lactating mice for 1−3 days (Spelford, Beijing, China) using 75% alcohol, the hearts were clipped. After washing in PBS, the hearts from lactating mice were transferred to a 50‐mL centrifuge tube containing 0.125% trypsin (Gibco, 25200072). Lactating mice hearts were digested overnight at 4°C. 0.8% Type II collagenase (Sigma, C2‐BIOC) was used to digest cardiac tissue in a 37°C water bath. All cells were resuspended and subjected to differential apposition for 1 h. The cells that adhered to the wall first were cardiac fibroblasts, which were cultured in DMEM containing 10% fetal bovine serum (FBS).

### 2.6 Immunofluorescence and immunohistochemistry

After fixing the heart tissue with 10% neutral formalin, paraffin‐embedded heart tissue was sectioned. Next, the paraffin sections underwent dewaxing and hydration, followed by antigen repair and closure. The primary antibody was diluted to 1:200 and incubated with the samples at 4°C overnight. On alternate days, after incubating samples with the fluorescent secondary antibody for 1 h at the room temperature, the sections were sealed with a DAPI‐containing sealer and observed under a fluorescent microscope. Immunohistochemical staining was performed using DAB and the brown precipitate was observed under light microscope.

### 2.7 Cellular transfection

Cardiac fibroblasts were seeded into six‐well plates and allowed to achieve a 50% cell density. After 24 h, the medium was exchanged. The transfection complexes were treated in accordance with the transfection reagent instructions (RiboBio, C10511‐05). The concentration of siRNA per well was 100 nmol. The transfection complexes were allowed to stand at room temperature for 15 min before being added to the six‐well plate. After 24‐h transfection, the medium was exchanged and the cells were exposed to hypoxic conditions.

### 2.8 Echocardiography

The mice were placed into an isoflurane anaesthesia machine for anaesthesia following hair removal. The small animal ultrasound machine was turned on, the mice were fixed on the operating table, the anaesthesia machine was connected and the heart rate of the mice was maintained between 400 and 500 beats per minute. The mouse was coated with coupling agent on the chest, the probe was adjusted to show the left ventricular long‐axis B‐mode image of the mouse, and the image was saved. The probe was rotated to reveal the short axis of the left ventricle, and the B‐image and M‐image were saved.^21^ Measurements were performed according to the M‐type image.

### 2.9 Statistical analysis

All data were presented as the mean ± SD (standard deviation). Data analysis was performed using GraphPad Prism 8 software. A one‐way ANOVA was used to analyze the data for three groups and above. The data from two groups were compared using Student's *t*‐test. A Mantel–Cox test was used to determine survival curves. *p*‐Values less than *.05* were considered to indicate a significant difference.

## 3 RESULTS

### 3.1 BMP6 expression is upregulated in a mouse myocardial infarction model

**FIGURE 1:**
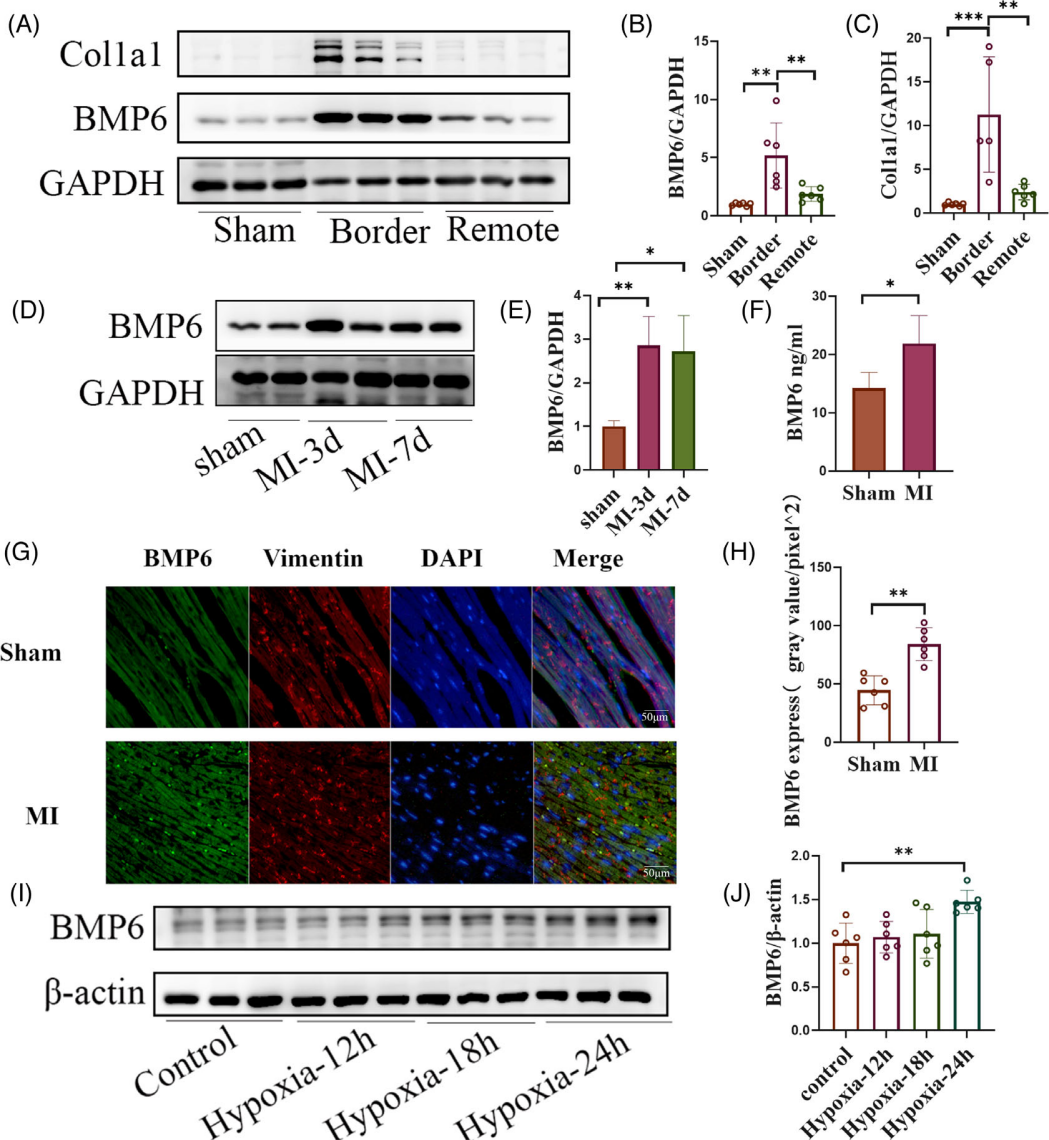
BMP6 expression is upregulated in a mouse myocardial infarction model. (A) The protein expression of BMP6 was detected from cardiac muscle by Western blot on day 14 post MI, which had original tissues from both border and remote of infarct region in wild‐type (WT) mice (*n* = 6). (B and C) Quantification of A‐plot results. (D) On the days 3 and 7 post MI, Western blot was used to detect the protein BMP6 in the infarct border zone (*n* = 6). (E) Quantification of D‐plot results. (F) The ELISA was used to detect serum BMP6 in sham‐operated group and MI group mice (*n* = 4). (G) Immunofluorescence detection of BMP6 expression after MI (heart section stained with an anti‐BMP6 is green, anti‐vimentin is red and DAPI is blue, scar bar: 50 μm (*n* = 6). (H) Quantification of G‐plot results. (I) The expression of BMP6 was detected from hypoxia‐induced cardiac fibroblasts using Western blot (*n* = 6). (J) Quantification of I‐plot results. All data are mean ± SD. One‐way ANOVA followed by Tukey's multiple comparisons test for (B), (C), (E) and (J). Student's *t*‐test for (F) and (H). Statistical significance was defined as *p < .05; **p < .01; ***p < .001.

A mouse myocardial infarction model was established to explore whether BMP6 plays a role in MI.^22^, ^23^ Western blot analysis detected BMP6 protein expression in the heart 14 days post MI. The findings revealed a significant upregulation of BMP6 protein expression in the marginal area of the MI group compared to the sham‐operated group (Figure 1A–C). In addition, we also detected the expression of BMP6 on the third and seventh day after MI. According to the results, the expression of BMP6 showed an upregulation trend post MI (Figure 1D,E). Meanwhile, ELISA result also indicated that serum BMP6 was upregulated post MI (Figure 1F). Subsequently, immunofluorescent double‐label staining was employed to detect the protein expression of BMP6, which yielded comparable findings (Figure 1G,H). In vitro, cardiac fibroblasts were extracted and cultured in a sugar‐free medium with a 2% oxygen concentration for 12, 18 and 24 h, respectively. Western blot results demonstrated that BMP6 expression was upregulated in response to anoxic incubation (Figure 1I,J). Taken together, these experimental findings provide evidence that BMP6 expression is upregulated following MI.

### 3.2 BMP6 knockout suppresses cardiac function after MI

**FIGURE 2:**
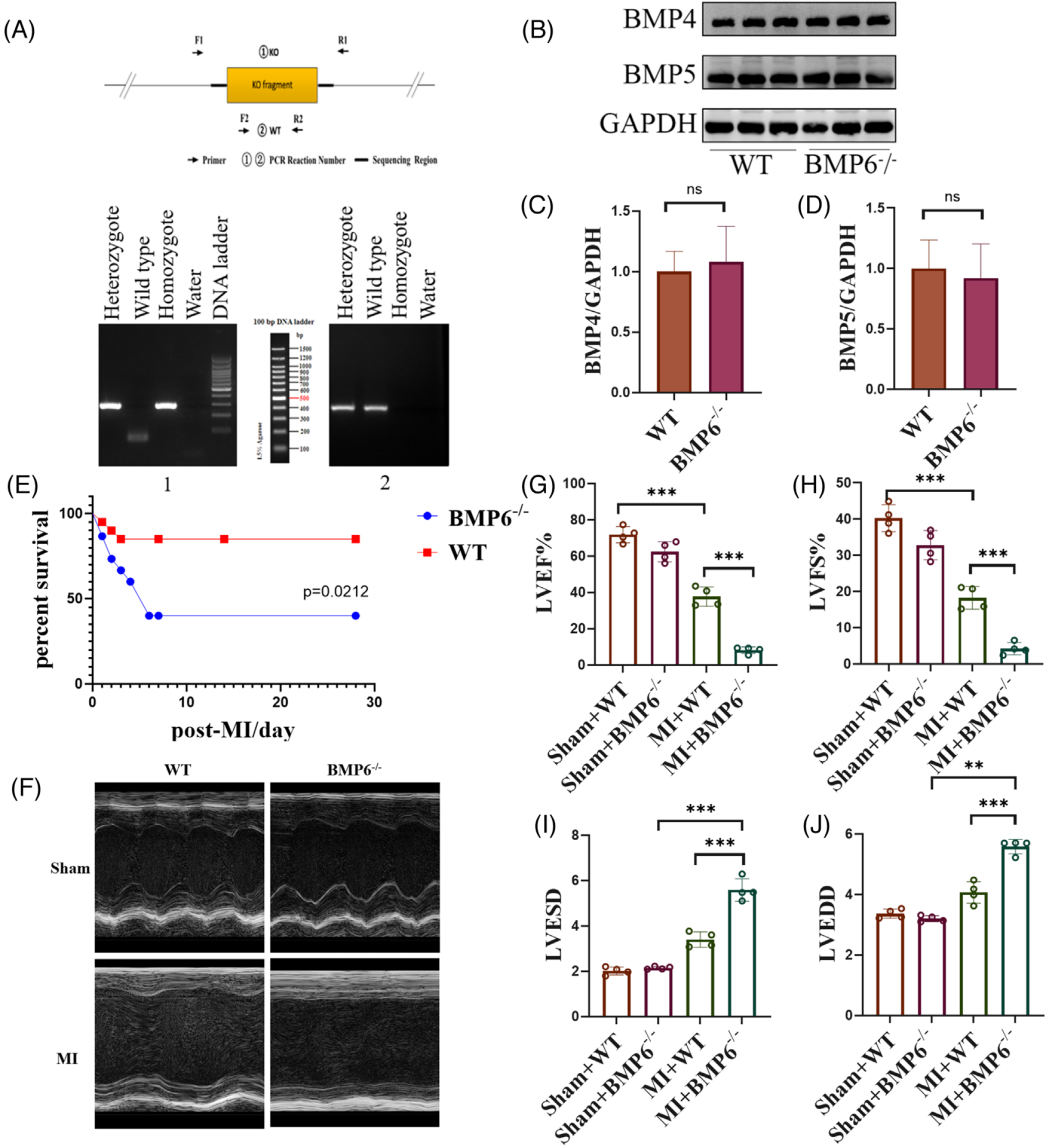
Knockout of BMP6 exacerbates cardiac failure after myocardial infarction. (A) Pattern map and genetic genotype identification results in BMP6^−/−^ mice. (B) The expression BMP4 and BMP5 by Western blot in WT and BMP6^−/−^ mice (*n* = 6). (C and D) Quantification of BMP4 and BMP5 protein expression. (E) Survival curves of WT mice and BMP6^−/−^ mice on day 28 post MI. (F) WT mice and BMP6^−/−^ mice were divided into sham‐operated and MI groups, with representative echocardiograms in different groups (*n* = 4). (G–J) WT mice and BMP6^−/−^ mice were divided into sham‐operated and MI groups, and the left ventricular ejection fraction (LVEF), left ventricular fractional shortening (LVFS), left ventricular end‐systolic diameter (LVESD) and left ventricular end‐diastolic diameter (LVEDD) of mice in each group. All data are mean ± SD. One‐way ANOVA followed by Tukey's multiple comparisons test for (G–J). Student's *t*‐test for (C) and (D). Mantel–Cox test for (E). Statistical significance was defined as *p < .05; **p < .01; ***p < .001; ns = not significant. WT: wild‐type.

To determine the role of BMP6 in MI, we deleted the BMP6 gene by CRISPR–Cas9 system to obtain BMP6^−/−^ mice (Figure 2A). After knocking out BMP6, we detected the protein expression of other members in the BMP family through Western blotting and found that knocking out BMP6 did not affect the protein expression of other members (Figure 2B–D). First of all, both BMP6 knockout and WT mice were divided into sham‐operated and MI groups, respectively. We plotted the survival curves of both the WT and BMP6 knockout mice for a period of 28 days after establishing an MI model. The findings indicate that the survival rate of BMP6 knockout mice after MI was significantly lower compared to that of the WT mice (Figure 2E). In BMP6 knockout mice, echocardiography revealed significantly impaired cardiac function following MI (Figure 2F). Compared with the sham‐operated group, the left ventricular ejection fraction (LVEF) and left ventricular fractional shortening (LVFS) were decreased in mice after MI. Nevertheless, the decrease in the LVEF and LVFS was more severe in the BMP6 knockout mice compared to the WT mice (Figure 2G,H). Correspondingly, left ventricular end‐systolic diameter (LVESD) and left ventricular end‐diastolic diameter (LVEDD) both increased in BMP6^−/−^ mice following MI (Figure 2I,J). The tentative results suggest that BMP6 might have a protective role in maintaining cardiac function following MI.

### 3.3 BMP6 knockout exacerbates myocardial fibrosis and inflammatory infiltration after MI

**FIGURE 3:**
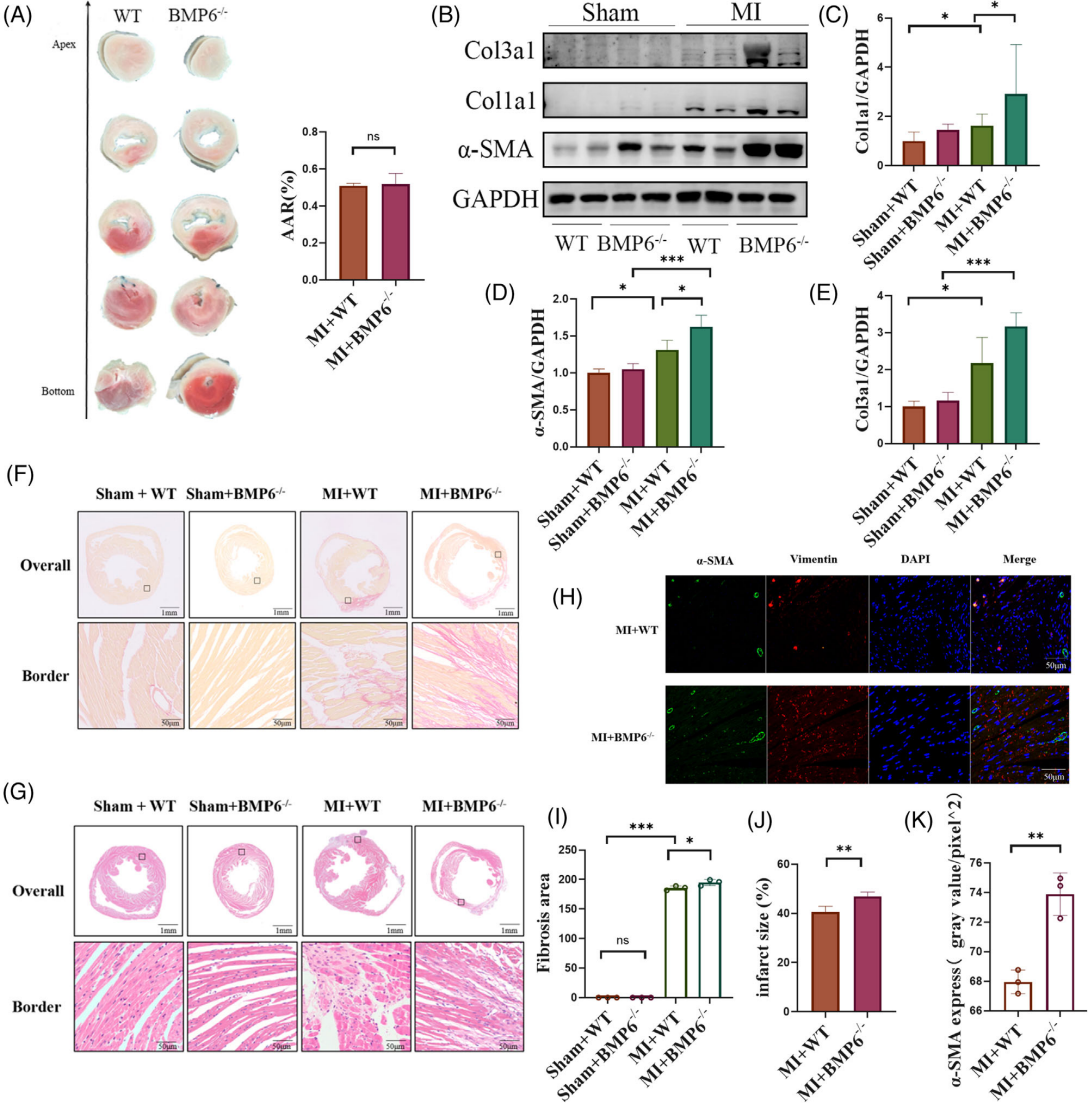
Knockout of BMP6 exacerbates myocardial fibrosis and inflammatory infiltration after myocardial infarction. (A) TTC staining 24 h post MI surgery in WT mice and BMP6^−/−^ mice (left panel); percentage of risk area after coronary artery ligation in mice (right panel) (*n* = 3). (B) To detect the protein expression of collagen on cardiac fibrosis by Western blot in WT mice, BMP6^−/−^ mice in sham and MI groups (*n* = 4). (C–E) Quantification of each protein expression. (F) Paraffin sections were subjected to Sirius red staining to detect cardiac collagen fibres from WT mice and BMP6^−/−^ mice, which were divided into sham and MI groups, respectively (collagen fibres in red, scar bar: 50 μm) (*n* = 3). (G) Paraffin sections were subjected to HE staining (scar bar: 50 μm) (*n* = 3). (H) Immunofluorescence detection of α‐SMA protein in different groups (scar bar: 50 μm) (*n* = 3). (I) Quantification of fibrosis area of heart in different groups. (J) Quantification of infarct size by ImageJ in heart. (K) Quantification of H‐plot results. All data are mean ± SD. One‐way ANOVA followed by Tukey's multiple comparisons test for (C–E) and (I). Student's *t*‐test for (A), (J) and (K). Statistical significance was defined as *p < .05; **p < .01; ***p < .001; ns = not significant.

To further determine the precise role of BMP6 in protecting cardiac function, TTC staining was performed 24 h after MI. The findings indicated that BMP6 did not bring about a significant change to the infarcted area of the heart during the acute stage of MI (Figure 3A). It was hypothesized that BMP6 may play a role in the process of ventricular remodelling. Based on the above assumptions, cardiac collagen deposition was examined 14 days post MI (Figure 3B). The findings indicate a notable increase in the expression of collagen I, collagen III and α‐SMA in the MI group compared to the sham group. However, the elimination of BMP6 exacerbated the deposition of cardiac collagen, leading to more severe consequences (Figure 3C–E). Furthermore, at 28 days post MI, Sirius red staining was performed to visualize collagen deposition. Based on our findings, we observed a more significant degree of myocardial fibrosis in the BMP6 knockout mice following myocardial infarction compared to the WT mice, which is consistent with previous results (Figure 3F,I). The HE staining results also revealed more severe inflammatory infiltration in BMP6 knockout mice after myocardial infarction (Figure 3G). The result in Figure 3J shows larger infarct size in BMP6 knockout mice. The immunofluorescence results showed an increased transition from fibroblasts to myofibroblasts after a knockout of BMP6 (Figure 3H,K). These experiments confirmed that BMP6 executed its effects to improve cardiac function after MI primarily by affecting inflammatory infiltration and ventricular remodelling.

### 3.4 In vitro knockdown of BMP6 exacerbates hypoxia‐induced collagen secretion by fibroblasts and their conversion into myofibroblasts

**FIGURE 4:**
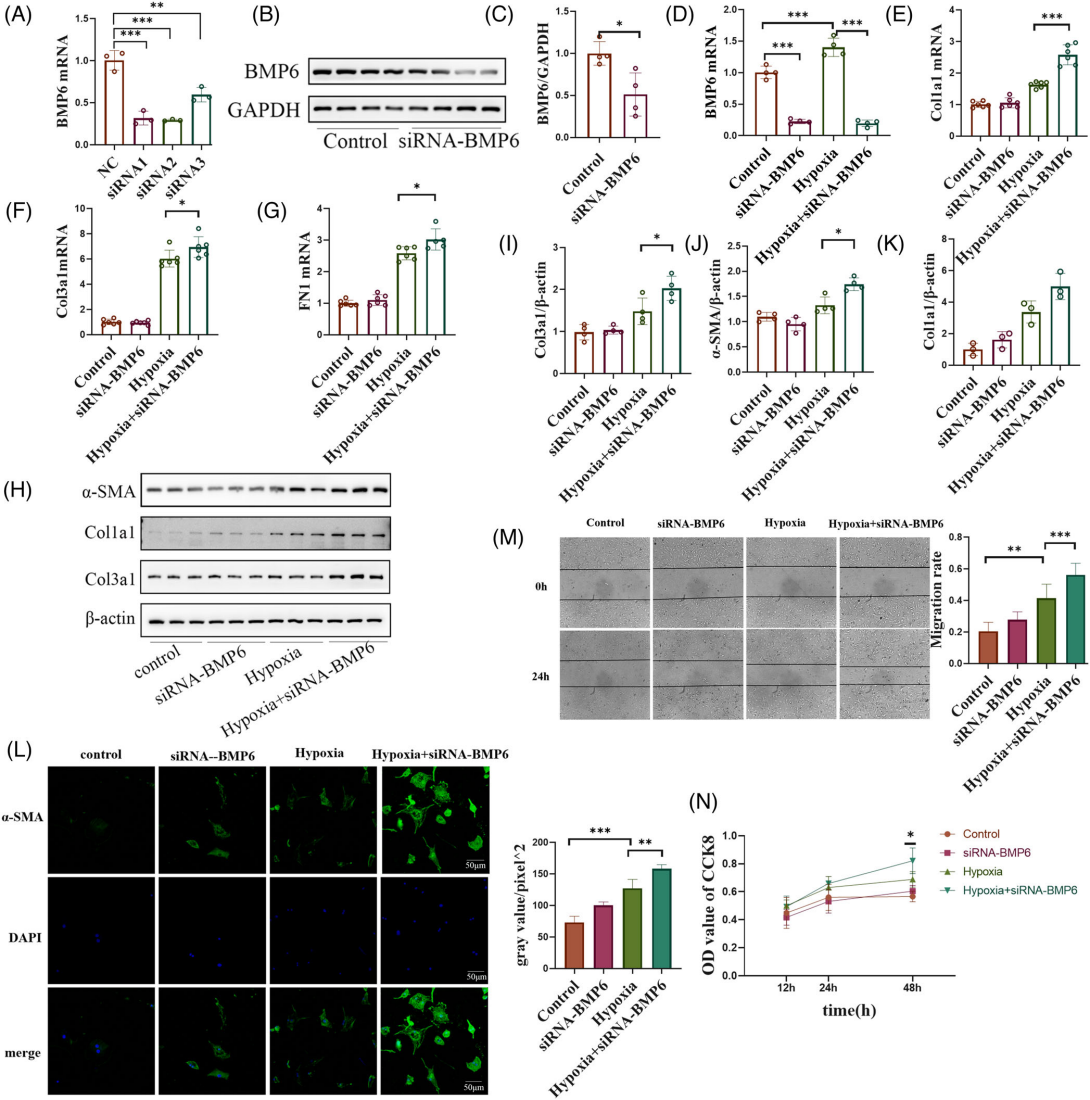
Knockdown of BMP6 exacerbates hypoxia‐induced injury in cardiac fibroblasts, which mainly promotes fibroblast migration, differentiation and proliferation. (A) In vitro, siRNA‐NC and siRNA‐BMP6 were transfected into cardiac fibroblasts, then we detected the mRNA expression of BMP6 (*n* = 3). (B) Verification of siRNA‐BMP6 knockdown at the protein level (*n* = 4). (C) Quantification of B‐plot results. (D–G) Fibroblasts were transfected with siRNA‐BMP6 following hypoxia state, and Q‐PCR was performed to detect the expression of BMP6, Col1a1, Col3a1 and FN1 mRNA in each group (*n* = 6). (H) Fibroblasts were transfected with siRNA‐BMP6 in hypoxia‐induced injury, and the expression of Col1a1, Col3a1 and α‐SMA protein was detected by Western blot (*n* = 4). (I–K) Quantification of H‐plot results. (L) Immunofluorescence staining the marker of α‐SMA in cardiac fibroblasts (*n* = 3) and quantification of L‐plot fluorescence staining results. (M) Cell scratching assay to detect fibroblast migration (*n* = 4) and quantification of M‐plot results. (N) CCK8 assay to detect fibroblast proliferation. All data are mean ± SD. One‐way ANOVA followed by Tukey's multiple comparisons test for (A), (D–G) and (I–N). Student's *t*‐test for (C). Statistical significance was defined as *p < .05; **p < .01; ***p < .001.

To validate the effect of BMP6 in vitro, primary cardiac fibroblasts were extracted from C57BL/6J lactating mice and transfected with siRNA. The Western blot and q‐PCR results showed that BMP6 expression was significantly downregulated after siRNA transfection (Figure 4A–C). As collagen deposition in the extracellular matrix is a key factor in the development of myocardial fibrosis, and fibronectin 1 (FN1) is involved in the promotion of collagen secretion by fibroblasts, we proceeded to investigate the expression of collagen I, collagen III and FN1. After knocking down BMP6 expression, hypoxia‐stimulated fibroblasts were used to simulate a myocardial infarction model in vitro. The q‐PCR results revealed an increase in collagen I, collagen III and FN1 mRNA expression after transfection with siRNA‐BMP6 relative to the hypoxia alone group (Figure 4D–G). Moreover, the Western blot results showed that collagen expression was markedly upregulated in cardiac fibroblasts following hypoxia, and a knockdown of BMP6 resulted in a more significant upregulation of collagen expression in fibroblasts (Figure 4H–K). Cellular immunofluorescence revealed an increase in differentiation of fibroblasts into myofibroblasts following a knockdown of BMP6 (Figure 4L). Furthermore, cell scratching assay also showed that a knockdown of BMP6 promoted fibroblast migration (Figure 4M). CCK8 experimental results demonstrated that silencing BMP6 promoted fibroblast proliferation (Figure 4N). Together, these results indicate that a knockdown of BMP6 promoted fibroblast proliferation, differentiation and migration, as well as upregulated collagen secretion by fibroblasts in vitro.

### 3.5 Overexpression of BMP6 in fibroblasts reverses hypoxia‐induced injury and reduces fibroblast overproliferation and migration

**FIGURE 5:**
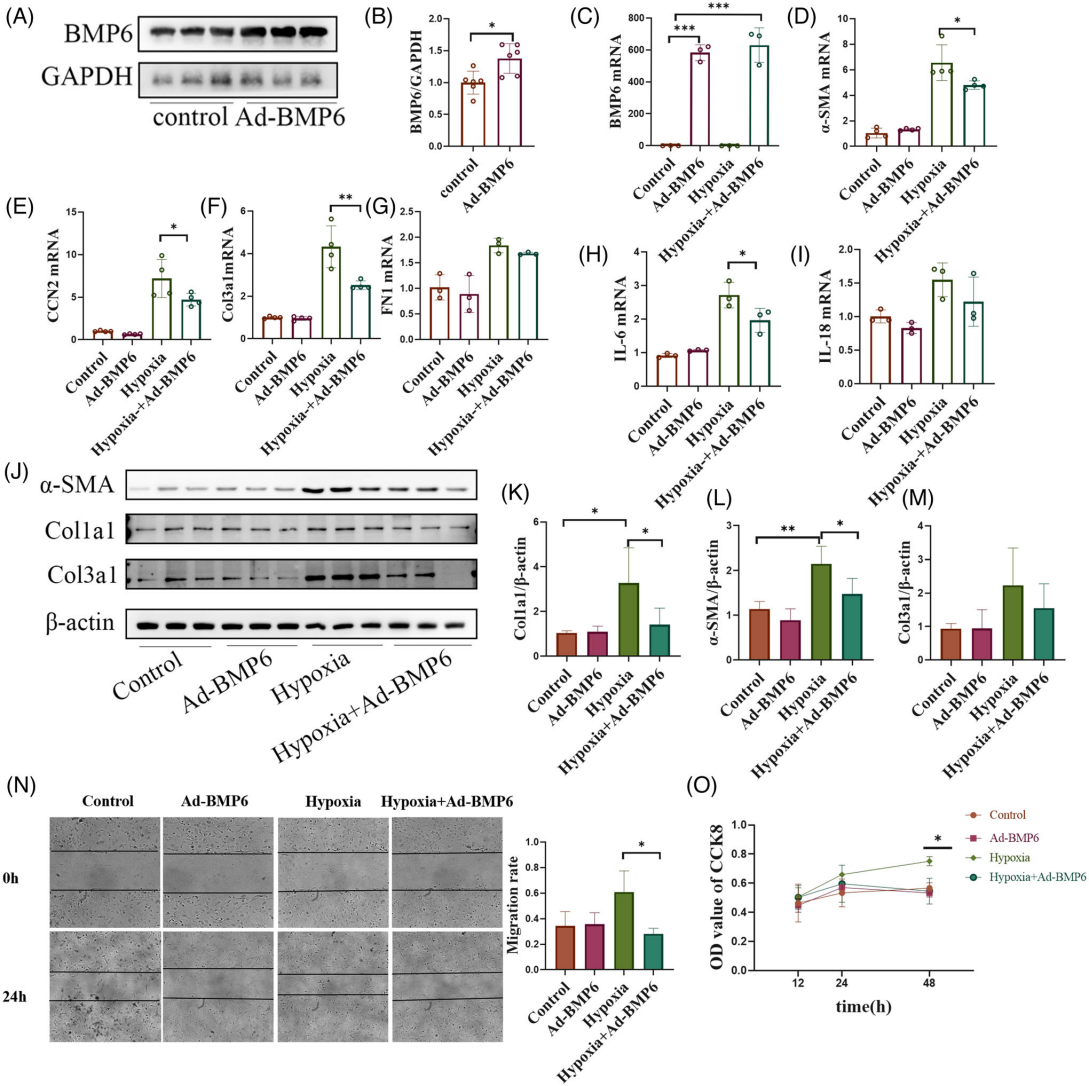
Overexpression of BMP6 reduces fibroblast over proliferation and migration in hypoxia‐induced cardiac fibroblasts. (A) Detection of BMP6 protein expression in fibroblasts after transfection with overexpressing adenovirus for 48 h (*n* = 6). (B) Quantification of A plot. (C–G) Detection of α‐SMA, Col3a1, FN1 and CCN2 mRNA level following overexpression of BMP6 in hypoxic state and anoxic state (*n* = 4). (H and I) After overexpression of BMP6, Q‐PCR was performed to detect the mRNA expression of IL‐6 and IL‐18 (*n* = 3). (J) The proteins of different collagens were detected by Western blot in hypoxic‐induced injuries from cardiac fibroblasts. (K–M) Quantification of J‐plot results. (N) After overexpression of BMP6, the results of cell migration in each group under hypoxic and anoxic conditions (left) and quantification of N‐plot in cell scratching experiments (right). (O) CCK8 assay to detect fibroblast proliferation. All data are mean ± SD. One‐way ANOVA followed by Tukey's multiple comparisons test for (C–I) and (K–O). Student's *t*‐test for (B). Statistical significance was defined as *p < .05; **p < .01; ***p < .001. Ad: adenovirus.

In the in vitro experiments, adenovirus‐mediated overexpression of BMP6 (Ad‐BMP6) in cardiac fibroblasts were performed to explore its function. The level of BMP6 expression was found to be significantly increased following Ad‐BMP6 infection (Figure 5A–C). To validate the role of BMP6, cardiac fibroblasts were cultured under hypoxic conditions after BMP6 overexpression. The q‐PCR results showed that the levels of collagen III, cellular communication network factor 2 (CCN2), FN1 and α‐SMA, myofibroblast marker, mRNA expression were upregulated following hypoxia stimulation. Nevertheless, BMP6 overexpression alleviated the pro‐fibrotic changes in hypoxic fibroblasts (Figure 5D–G). CCN2 is a pro‐fibrotic cytokine that is involved in the process of myocardial fibrosis. FN1 plays a role in promoting collagen secretion by fibroblasts. The q‐PCR results also revealed downregulation of the expression of the inflammatory factors IL6 and IL18, following BMP6 overexpression, indicating that BMP6 may inhibit the secretion of inflammatory factors after hypoxia (Figure 5H,I). We also examined the levels of collagen I, collagen III and α‐SMA protein expression by Western blot (Figure 5J–M), and obtained similar results. The cell scratch assay also showed that BMP6 could reduce fibroblast migration (Figure 5N). CCK8 experimental results illustrated that overexpression of BMP6 inhibited fibroblast proliferation (Figure 5O). Therefore, loss‐of‐function and gain‐of‐function were used to doubly verify that BMP6 inhibited cardiac fibrosis progression and inflammatory infiltration in cardiac fibroblasts after hypoxia in vitro.

### 3.6 RNA‐seq indicates that CEMIP is involved in ventricular remodelling regulation by BMP6

**FIGURE 6:**
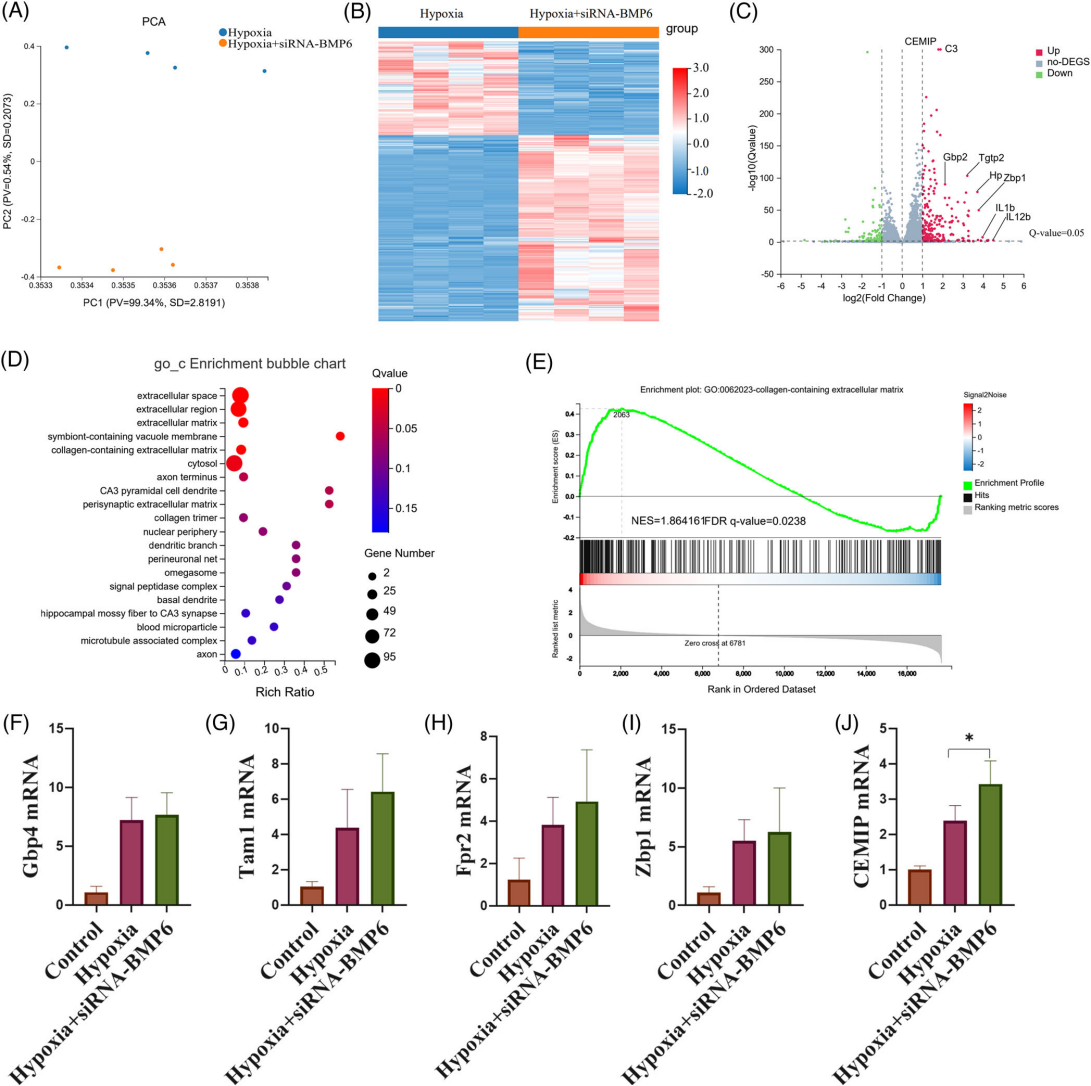
RNA‐seq indicates that cell migration‐inducing protein (CEMIP) is involved in the regulation of ventricular remodelling by BMP6. (A) PCA of hypoxic group with hypoxia+siRNA‐BMP6 composed of fibroblasts. (B) Heatmap of differential genes in the hypoxic and hypoxic+siRNA‐BMP6 groups. (C) A volcano plot of differentially expressed genes in the hypoxia group versus the hypoxia+siRNA‐BMP6 group is shown, with upregulated genes in the red, and downregulated genes in the green (*n* = 4). (D) GO‐C enrichment for differential genes. (E) GSEA analysis diagram of the relationship between BMP6 knockdown and genes related to cardiac fibrosis under the situation of hypoxia. (F–J) Analysis of mRNA of Gbp4/Tam1/Fpr2/Zbp1/CEMIP gene in different groups (*n* = 3). All data are mean ± SD. One‐way ANOVA followed by Tukey's multiple comparisons test for (F–J). Statistical significance was defined as *p < .05; **p < .01; ***p < .001.

To investigate the potential mechanism associated with BMP6 regulation of myocardial fibrosis, we extracted cardiac fibroblasts from lactating mice and transfected them with BMP6 siRNA and scramble siRNA. Next, RNA‐seq was performed on fibroblasts after hypoxia. The results showed that there were 345 differentially expressed genes following a BMP6 knockdown (230 upregulated and 115 downregulated genes) (Figure 6A–D). As expected, some pro‐fibrotic genes were significantly upregulated. GSEA analysis showed that silencing BMP6 was associated with extracellular matrix secretion (Figure 6E). Next, we detected the levels of mRNA expression of the above genes, and eventually cell migration‐inducing protein (CEMIP) was selected as the downstream target (Figure 6F–J). A previous study indicated that CEMIP induced chondrocyte fibrosis in osteoarthritis.^24^ Anti‐CEMIP antibodies have also been found to inhibit renal fibrosis in obese patients by inhibiting the Wnt/β‐catenin pathway,^25^ and CEMIP inhibition inhibited fibroblast proliferation and differentiation.^26^ Therefore, we speculate that CEMIP may represent a potential mechanism through which silencing BMP6 expression can aggravate myocardial fibrosis.

### 3.7 BMP6 regulates myocardial fibrosis via AP‐1/CEMIP

**FIGURE 7:**
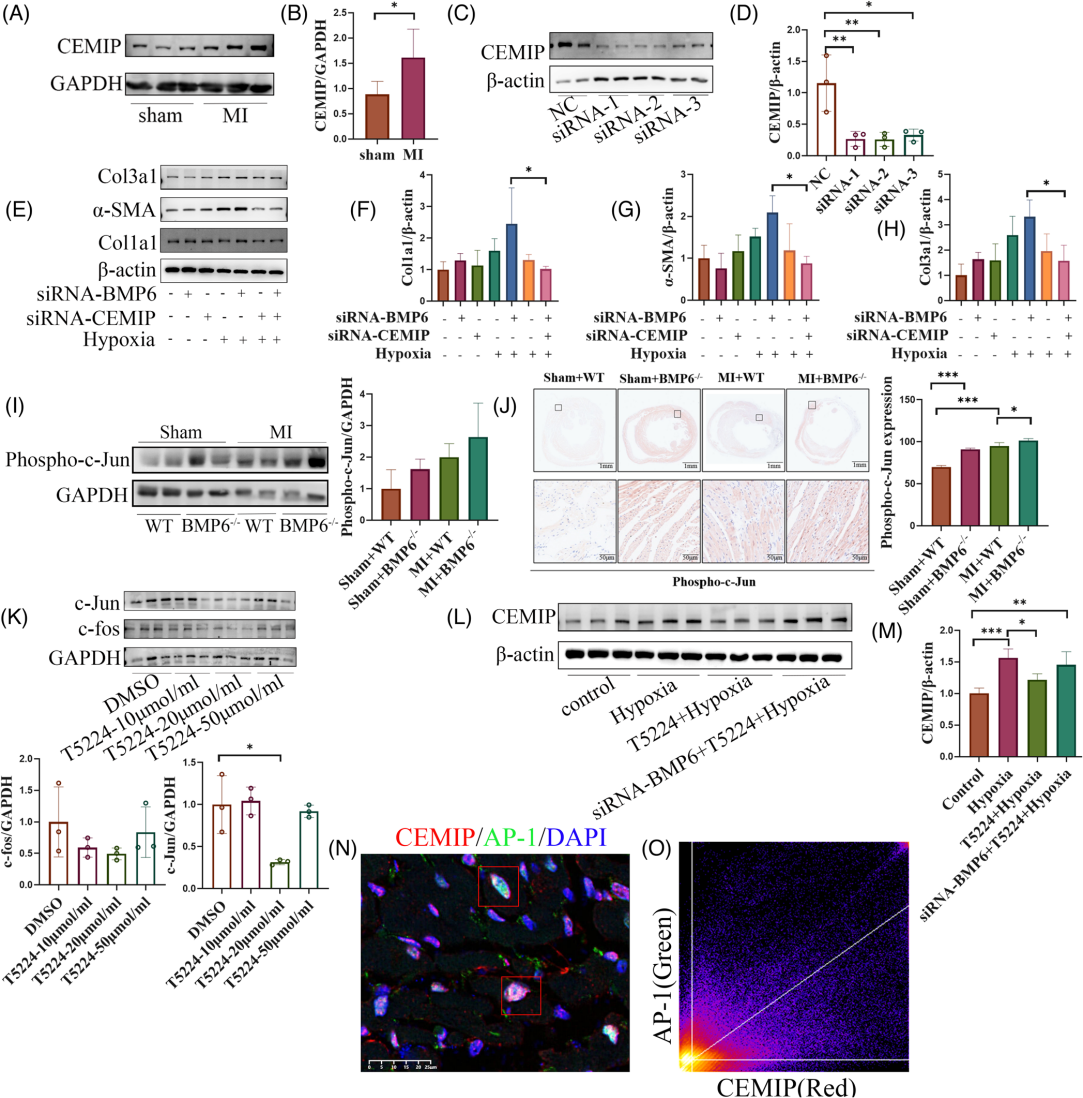
BMP6 regulates CEMIP through c‐Jun phosphorylation to improve myocardial fibrosis. (A) The protein level of CEMIP was detected by Western blot post MI (*n* = 6). (B) Quantification of A‐plot results in the different groups. (C) To verify the results of siRNA‐CEMIP by Western blot (*n* = 4). (D). Quantification of C‐plot results. (E) To detect collagen expression by Western blot in cardiac fibroblasts under different conditions (*n* = 3). (F–H) Quantification of E‐plot results. (I) Expression of phospho‐c‐Jun in WT mice versus BMP6 knockout mice in different groups (*n* = 3). (J) Immunohistochemical detection of phospho‐c‐Jun expression in different groups. (K) Expression results of c‐fos, c‐Jun after T5224 stimulation of fibroblasts and the quantification of K‐plot results. (L) Expression of CEMIP under different states in cardiac fibroblasts (*n* = 3). (M) Quantification of L‐plot results. (N) Immunofluorescence staining for co‐localization of AP‐1/CEMIP. (O) Quantification of N‐plot. All data are mean ± SD. Student's *t*‐test for (B). One‐way ANOVA followed by Tukey's multiple comparisons test for (D), (F–K) and (M). Statistical significance was defined as *p < .05; **p < .01; ***p < .001.

First, the protein expression level of CEMIP was detected by Western blotting following myocardial infarction. The results showed that CEMIP expression was upregulated post MI (Figure 7A,B). To clarify whether CEMIP inhibition represents a key mechanism by which BMP6 exerts its inhibitory effect on fibrosis, related experiments were performed using CEMIP siRNA to silence CEMIP expression. Figure 7C,D shows that CEMIP protein expression was significantly downregulated. Fibroblasts were transfected with siRNA‐BMP6, siRNA‐CEMIP, and co‐transfected before hypoxia treatment, respectively. The Western blot results showed that the expression of collagen I, collagen III and α‐SMA was upregulated in the transfected siRNA‐BMP6 group compared to the hypoxic group alone. Moreover, both the co‐transfection and transfection of siRNA‐CEMIP alone alleviated the increased collagen secretion caused by a knockdown of BMP6 (Figure 7E–H). Therefore, these results indicate that BMP6 may regulate the ventricular remodelling process following MI through CEMIP.

It has been shown that CEMIP expression in breast cancer is dependent on the AP‐1 binding site in the promoter region.^27^ Moreover, a knockout of BMP6 can promote the transcriptional activity of AP‐1.^15^ In the present study, we found that AP‐1 phosphorylation was increased following a knockdown of BMP6 (Figure 7I) and similar results could be obtained from immunohistochemical results (Figure 7J). Consequently, it is reasonable to suspect that BMP6 inhibited CEMIP expression by suppressing AP‐1 transcriptional activity, thereby slowing the progression of myocardial fibrosis after MI. The AP‐1 inhibitor, T5224, was used to silence AP‐1 expression, set at 10, 20 and 50 μm/mL.^28^ The results showed that the best silencing effect was achieved at a concentration of 20 μm/mL (Figure 7K). The hypoxic cardiac fibroblasts were divided into hypoxia‐only group, hypoxia+T5224 group and hypoxia+siBMP6+T5224 group. According to the experimental results (Figure 7L,M), CEMIP expression was upregulated after hypoxic stimulation, whereas an AP‐1 inhibitor could inhibit CEMIP expression under hypoxic conditions. Subsequently, immunofluorescence staining results showed colocalization of AP‐1 with CEMIP in myocardial tissue (Figure 7N,O). The above observations indicate that silencing BMP6 could exacerbate myocardial fibrosis by promoting AP‐1 phosphorylation, upregulating its transcriptional activity and thus CEMIP expression, which in turn exacerbates myocardial fibrosis.

### 3.8 Recombinant human protein BMP6 reduces myocardial fibrosis and improves cardiac function after MI

**FIGURE 8:**
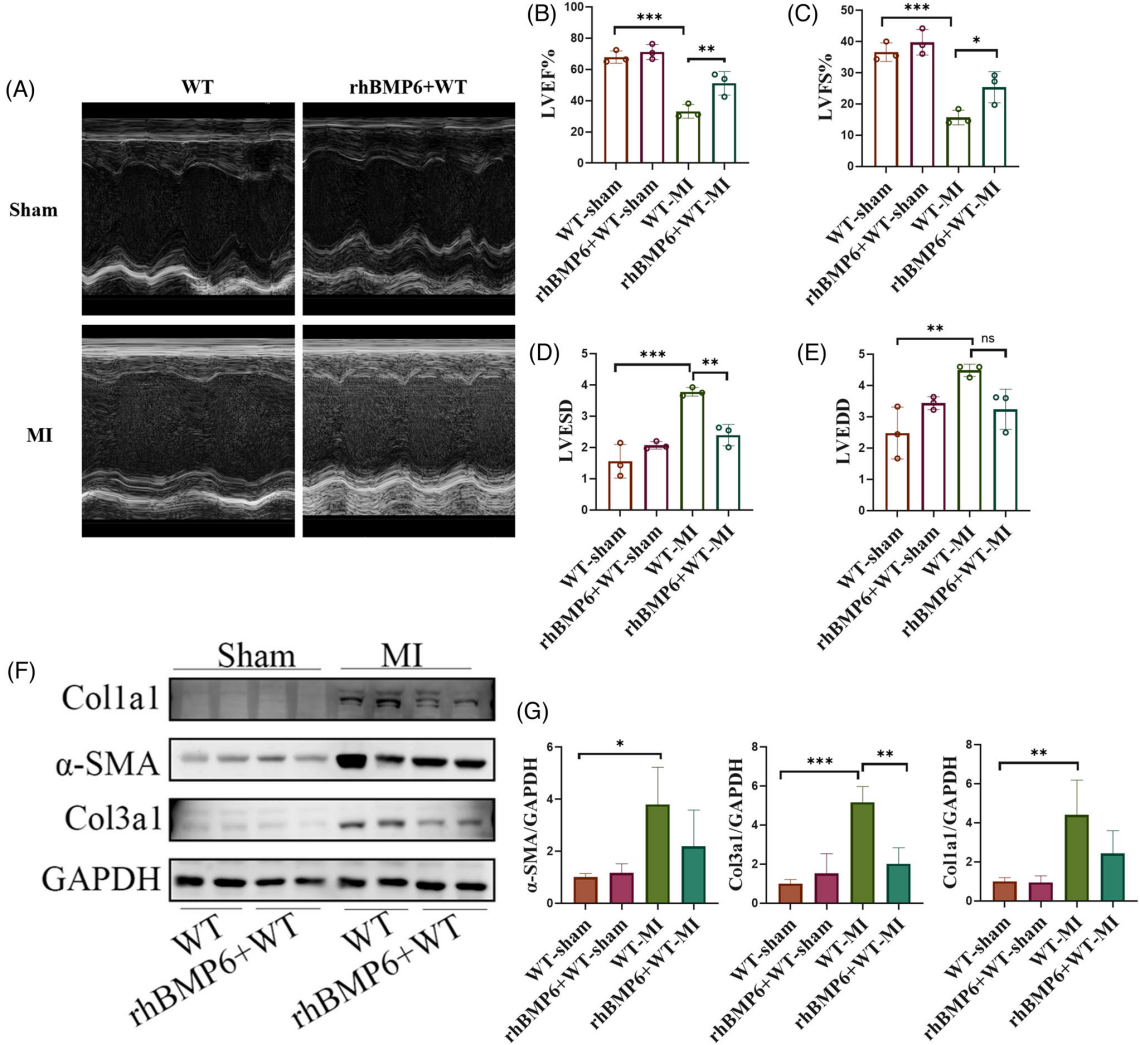
Recombinant human protein BMP6 (rhBMP6) enhances cardiac function after myocardial infarction in WT mice. (A) The WT mice were divided into two groups: WT mice alone and WT mice + rhBMP6 tail vein administration group. The echocardiograms of each group were obtained after creating models of MI or conducting sham procedures on mice. (B–E) LVEF, LVFS, LVESD, LVEDD in different groups of mice (*n* = 3). (F) To detect the collagen deposition by Western blot in MI mice (*n* = 3). (G) Quantification of F‐plot results. All data are mean ± SD. One‐way ANOVA followed by Tukey's multiple comparisons test for (B–E) and (G). Statistical significance was defined as *p < .05; **p < .01; ***p < .001.

Previous studies have shown that BMP6 has a protective function in ventricular remodelling. However, its clinical translational significance remains unknown. After establishing MI in WT mice, a tail vein injection of recombinant human BMP6 protein (rhBMP6) was administered. Echocardiography (Figure 8A) showed that treatment with rhBMP6 alleviated the reduction in LVEF, mitigated the decrease in LVFS and prevented the increase in LVESD and LVEDD in mice after MI (Figure 8B–E). The level of collagen I, collagen III and α‐SMA expression in the infarct marginal zone of heart tissue after myocardial infarction in mice was detected by Western blot. The results showed that treatment with rhBMP6 reduced collagen deposition and the conversion of fibroblasts to myofibroblasts after MI (Figure 8F,G). The findings suggest that rhBMP6 may alleviate myocardial fibrosis and improve cardiac function following MI, thus providing a promising rationale for the clinical translation of this therapy.

## 4 DISCUSSION

In other organ tissues, fibroblasts secrete collagen following proliferation and differentiation to promote wound healing, and their secreted collagen fibres can subsist on their own at a later stage.^29^ This prevents organs from becoming stiff and adopting a non‐contractile elastic condition. Following the ventricular remodelling, cardiac fibrosis is irreversible and maintained, resulting in a state of stiffness and solidification, loss of contractile elasticity and dysfunction of the heart tissue. These changes may eventually lead to ventricular rupture causing death. Hence, a reduction of myocardial fibrosis during ventricular remodelling is essential for a favourable prognosis following MI, during which more secretion of collagen after proliferation and differentiation of fibroblasts is the key biological activity.^30^

In previous studies, the role of BMP6 was primarily investigated in the regulation of iron^31^ and tumours.^11^, ^12^, ^32^ In this study, we focused on the role of BMP6 in myocardial fibrosis during ventricular remodelling following MI. Our results showed that BMP6 protein expression was upregulated after MI, which was consistent with a clinical study that found elevated BMP6 expression among the sera of patients with advanced heart failure.^10^ This was also observed in liver and renal fibrosis.^13^, ^33^ The serum BMP6 was upregulated, indicating BMP6 function as a marker for predicting myocardial infarction. Interestingly, BMP6 can also be secreted into the circulation by the liver.^34^ Evidence suggests that BMP receptors are present in endothelial cells, indicating that BMP6, secreted by the liver into the circulation, may play a role in regulating cardiac function by binding to receptors in vascular endothelial cells.^35^ Furthermore, research has demonstrated that BMP6 has the ability to regulate angiogenesis, indicating that BMP6 may have a functional pathway in the circulation.^36^ Angiogenesis plays an important role in ventricular remodelling after myocardial infarction.^37^ Liver‐released BMP6 may also affect cardiac function, which may be related to angiogenesis. The role of liver‐released BMP6 on cardiac function deserves further exploration in future studies. In addition, in vivo experiments indicated that BMP6 plays a significant role in the long‐term survival of mice post MI. Moreover, BMP6 could protect cardiac function in mice after MI. Similar effect of BMP6 was reported in a rat model of cerebral ischaemia.^38^ Besides, a knockout of BMP6 reduced myocardial fibrosis, which was in line with the results reported.^13^, ^39^ Although these studies were based on the fact that BMP6 deficiency exacerbates renal fibrosis and liver fibrosis, the results are consistent for BMP6‐mediated inhibition of fibrotic state in different organs.

**FIGURE 9:**
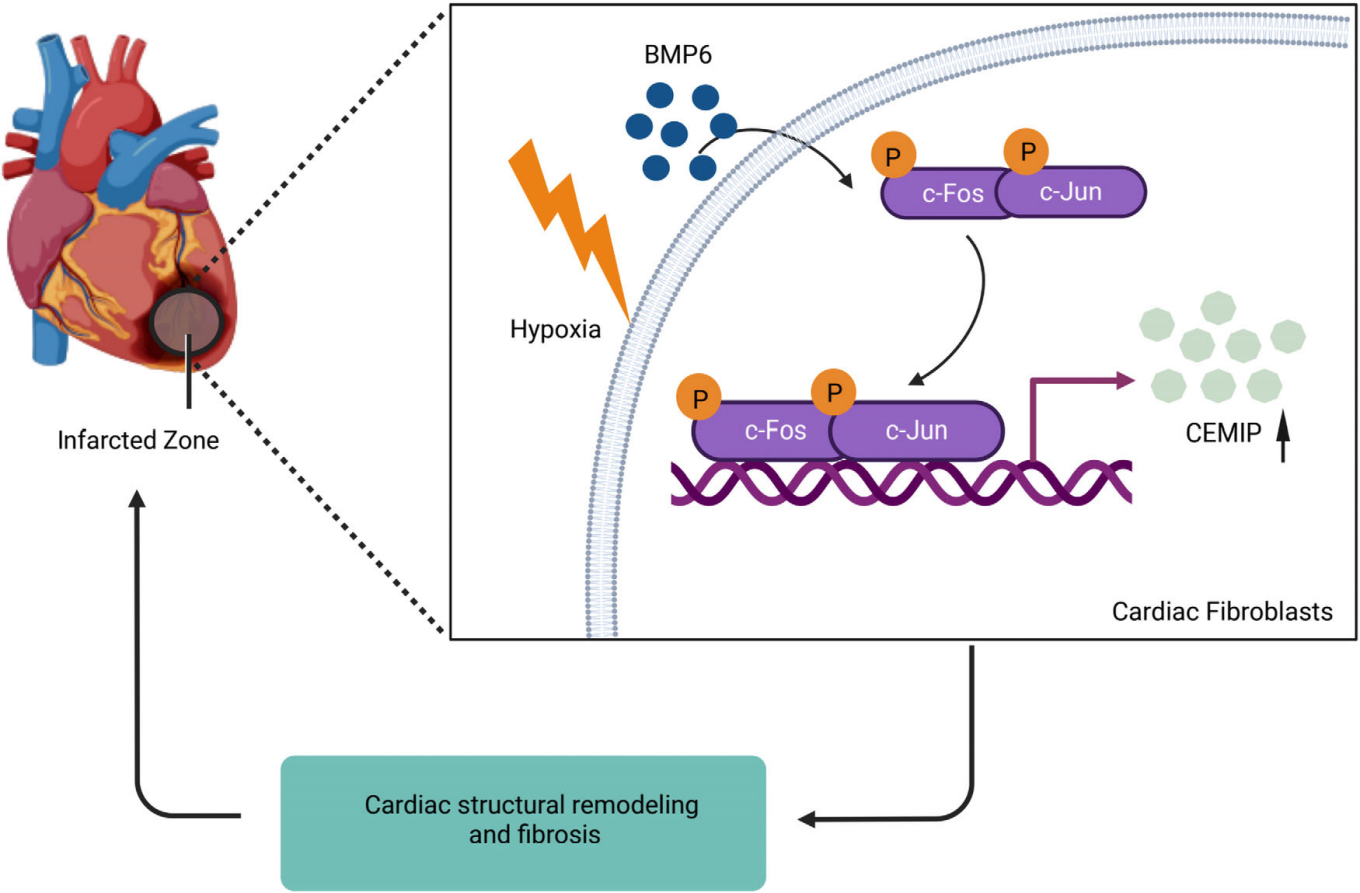
Mechanism of BMP6 regulates myocardial fibrosis through AP‐1/CEMIP. • Knocking out BMP6 promotes AP‐1 phosphorylation in hypoxia‐induced cardiac fibroblasts. • The expression of CEMIP is upregulated post MI, which is affected by AP‐1/BMP6. • rhBMP6 affects the process of cardiac remodelling, which may be a potential therapeutic target in the cardiac remodelling post MI.

One of the hallmarks of myocardial fibrosis is the transformation of myofibrils and increased extracellular matrix collagen secretion.^30^ In this process, fibroblasts are the main source of myofibroblasts.^40^ In vitro, our study used gain‐of‐function and loss‐of‐function experiments to clarify the role of BMP6 in fibroblasts, and found that BMP6 reduces collagen secretion by suppressing fibroblast proliferation. These results are similar to previous reports that showed that a knockdown of BMP6 could also promote fibrosis in other organs.

To clarify the mechanism by which BMP6 regulates myocardial fibrosis, lactating mice cardiac fibroblasts were extracted before a transfection with BMP6. Then the RNA‐seq was performed. Among the differentially expressed genes, the upregulation of CEMIP was our focus. CEMIP is a cell migration‐inducing protein that is primarily associated with cancer cell migration^41^ and has been shown to regulate the proliferation and differentiation of fibroblasts.^26^ As regards to experiment result, collagen expression was upregulated after silencing the BMP6 as expected. However, the upregulation of collagen secretion achieved by silencing BMP6 could be reversed by co‐silencing the expression of CEMIP. Similar to our findings, Deroyer et al. reported that CEMIP induced a fibrosis‐like process in osteoarthritic chondrocytes,^24^ and Chen et al. showed that anti‐CEMIP antibodies can inhibit renal fibrosis.^25^ These findings tentatively suggest that BMP6 can inhibit the expression of CEMIP, thereby inhibiting extracellular matrix deposition and thus protecting cardiac function.

Enhanced AP‐1 transcriptional activity plays an important role in the regulation of fibrotic diseases.^42^, ^43^, ^44^ Moreover, it has been found that BMP6 can inhibit the transcriptional activity of AP‐1, thereby functioning as a suppressor of skin fibrosis.^15^ And a significant upregulation of AP‐1 expression was observed following a knockout of BMP6 after MI. Our results are consistent with the results of study from Arndt et al, which reported increased AP‐1 phosphorylation and enhanced activity after a knockout of BMP6. Additionally, AP‐1 was also associated with the expression of CEMIP.^27^ Therefore, the co‐stimulation of fibroblasts with the AP‐1 inhibitor, T5224, and siRNA‐BMP6 resulted in upregulation of CEMIP after hypoxia. This expression was downregulated after treatment with the AP‐1 inhibitor, whereas treatment with siRNA‐BMP6 could reverse the results induced by T5224. In our study, we have preliminarily demonstrated that AP‐1 inhibitors can be used to inhibit CEMIP expression, providing new insight into the mechanisms associated with upregulation of AP‐1 expression following knockdown of BMP6, which in turn causes CEMIP upregulation and thus exacerbates ventricular remodelling after MI.

There are several potential clinical therapeutic roles for rhBMP6. The study by Yan et al. found that rhBMP6 could reduce the expression of collagen.^14^ In this study, we also investigated the specific function of rhBMP6 in cardiac fibrosis, which was shown to delay the decline of cardiac function and reduce extracellular matrix deposition in previous reports.

Collectively, our findings indicate that cardiac dysfunction worsens after a BMP6 knockout. Moreover, knockdown of BMP6 aggravates myocardial fibrosis after MI mainly through upregulating the expression of AP‐1/CEMIP (Figure 9). Consequently, our research may provide a novel therapeutic target for cardiac fibrosis during MI.

## CONFLICT OF INTEREST STATEMENT

The authors declare they have no conflicts of interest.
